# Zoonotic Enteric Nematodes and Dermatophytes in Cat Cafés: An Investigation in the Bangkok Metropolitan Area, Thailand

**DOI:** 10.3390/vetsci11080358

**Published:** 2024-08-07

**Authors:** Phakjira Sanguansook, Siwaporn Tuangpermsub, Boonyakorn Leelakarnsakul, Sutida Phaisansomsuk, Vachira Hunprasit, Laura Del Río, Waree Niyomtham, Nuvee Prapasarakul, Woraporn Sukhumavasi

**Affiliations:** 1 Bachelor Degree Program, Faculty of Veterinary Science, Chulalongkorn University, Bangkok 10330, Thailand; 2 Department of Veterinary Medicine, Faculty of Veterinary Science, Chulalongkorn University, Bangkok 10330, Thailand; 3 Department of Animal Health, School of Veterinary Sciences, University of Murcia, Campus de Espinardo, 31000 Murcia, Spain; laurario@um.es; 4 Department of Veterinary Microbiology, Faculty of Veterinary Science, Chulalongkorn University, Bangkok 10330, Thailandnuvee.p@chula.ac.th (N.P.); 5 Center of Excellence in Diagnosis and Monitoring of Animal Pathogens, Chulalongkorn University, Bangkok 10330, Thailand; 6 Parasitology Unit, Department of Pathology, Faculty of Veterinary Science, Chulalongkorn University, Bangkok 10330, Thailand; 7 Feline Infectious Disease and Health for Excellence Research Unit, Microbial Food Safety and Antimicrobial Resistance Research Unit, Center of Excellence in Animal Vector-Borne Diseases, Chulalongkorn University, Bangkok 10330, Thailand

**Keywords:** Bangkok, cat café, dermatophyte, gastrointestinal parasites, zoonosis

## Abstract

**Simple Summary:**

The cat café has emerged as a popular and growing business in Thailand, allowing customers to have direct contact with cats, a species known to harbor certain zoonotic diseases. Given the previous reports of zoonotic parasites and fungal pathogens in the cat population in Thailand, there is a potential for these pathogens to be present in cat cafés. The objective of this study is to investigate the prevalence of potentially zoonotic enteric parasites and dermatophytes in cat cafés in Bangkok and its vicinity. Out of 11 cat cafés, zoonotic enteric nematodes composed of hookworm and roundworm were identified in 2 cafés. Furthermore, fungal cultures of individual cat hair samples indicated that 16.2% (32/198) of the cats were positive for dermatophytosis. In summary, zoonotic parasites and dermatophytes were detected in cat cafés in Bangkok and the surrounding provinces. The evidence based on the prevalence and risk factors associated with these pathogens will heighten awareness among stakeholders and customers involved in this business.

**Abstract:**

Cat cafés have gained significant popularity worldwide, offering a unique interface between humans and cats. The present study aims to assess the prevalence of potentially zoonotic endoparasites and dermatophytes from cats living in cat cafés situated in the Bangkok metropolitan area in 2017–2018. Cat fecal samples were subjected to microscopic examination employing centrifugal flotation and centrifugal sedimentation techniques. The hair samples from every cat were cultured on a dermatophyte test medium and Sabouraud dextrose agar and subsequently confirmed by visualization of the typical colony and macroconidia morphology. Findings from 11 cat cafés indicated an 18.2% (2/11) prevalence of gastrointestinal parasites, including *Toxocara* spp., *Ancylostoma* spp., *Physaloptera* spp., and *Eucoleus aerophilus*. Dermatophytes were prevalent in 16.2% (32/198) of the total number of cats tested, with *Microsporum canis* being the sole species identified. Notably, the presence of dermatophyte was significantly correlated with the presence of skin lesions and the cats’ origin. In summary, the findings of this study have provided evidence of potentially zoonotic endoparasites and dermatophytes in cats residing in cat cafés. Therefore, it is imperative to heighten awareness and encourage preventive measures among cat café owners and customers to halt the dissemination of these pathogens.

## 1. Introduction

Cat cafés, an emerging business model within Thailand’s tourism industry, have surged in popularity, attracting both local and international tourists. The growth of such establishments in Thailand has been considerable since 2014 [1], predominantly in urban areas, such as Bangkok. These cafés cater to the needs of apartment residents unable to keep pets in their homes or simply provide a haven for cat lovers to visit [2]. Patrons at these establishments are typically allowed to observe, feed, and interact with the resident cats while partaking in meals, desserts, and beverages. Essentially, people can pet cats while they eat and drink [3].

Zoonotic transmission from cats to humans can occur through several mechanisms. Firstly, zoonotic gastrointestinal parasites can be spread by the fecal–oral route. Humans can be infected with pathogens from genera such as *Toxocara*, *Toxoplasma*, *Cryptosporidium*, and *Giardia* by consuming food or water contaminated with parasite eggs, oocysts, or cysts [4,5,6,7]. Notably the latter two pathogens are capable of being immediately infectious upon excretion into feces [5,6]. The accidental ingestion of infected vectors from contaminated food can also serve as a transmission route for zoonotic pathogens such as *Dipylidium caninum* and *Physaloptera* spp. [4,8,9]. Additionally, certain gastrointestinal parasites can cause skin larval migration through direct contact with soil, particularly those from the genera *Ancylostoma* and *Strongyloides* [4,10]. A study in Japan identified the risk of human infection with *Giardia duodenalis* from cats in cafés and shelters, finding zoonotic *G. duodenalis* (assemblage A) in two cat cafés via PCR analysis [11]. There was also a report of gastrointestinal parasites in clinically healthy cats in Bangkok and its vicinities, showing a high prevalence of *Ancylostoma* spp. and other potentially zoonotic endoparasites such as *Toxocara cati*, *Dipylidium caninum*, *Eucoleus aerophilus*, *Strongyloides* spp. and *Giardia* spp. [12]. However, no comparable studies have been conducted on cat cafés population in Thailand.

Dermatophytosis, a common contagious skin disease in various animal species and humans, also poses a significant risk. *Microsporum canis* is the most common cause of feline dermatophytosis [13] with cats serving as a major reservoir [14,15]. Most infected cats are asymptomatic or display mild clinical signs such as patchy areas of short stubble, alopecia, scales, or erythematous plaques, and humans can contract dermatophytes through direct contact [16,17]. Particular caution is necessary to prevent infection for immunocompromised individuals [18]. Previous studies highlighted the prevalence of asymptomatic cats harboring dermatophytes within veterinary hospitals and cat farms in Thailand [17,19]. However, no studies have yet been conducted within cat cafés in Thailand.

Given the potential for zoonotic transmission of parasites and dermatophytes from cats in these establishments, the aim of this study was to determine the prevalence of potentially zoonotic enteric nematodes, *Giardia* and dermatophytes, in cat cafés in the Bangkok metropolitan area. Furthermore, this study sought to examine individual factors and management practices that may pose additional risks.

## 2. Materials and Methods

### 2.1. Study Population and Sample Collection

The present study was conducted on cats residing in cat cafés within the metropolitan area of Bangkok. Data regarding the age, sex, breed, and origin of each individual cat were obtained through interviews with café staff and observation of skin lesions during sample collection. Utilizing a cross-sectional study design, a convenience sampling method was employed, whereby 11 out of the 21 cat cafés operating in 2017 in Bangkok and the surrounding metropolitan area were included. The period of sample collection extended from August 2017 to March 2018.

To evaluate the prevalence of endoparasites from 11 cat cafés, a total of 268 fresh fecal samples were directly collected from litter boxes over two consecutive days to ensure comprehensive coverage of all cats in each café, based on the understanding that the majority of healthy cats defecate at least once a day [20]. To evaluate the level of parasite problem in each café, the percentages of endoparasite-positive rate from each café were calculated from the number of positive samples from each café and the total number of tested samples. Additionally, on the same day of the cat café fecal sample collection, at least 3 fecal samples from free-roaming outdoor and stray cats living in a Thai Buddhist temple were collected as a positive control for fecal flotation and sedimentation techniques. Fecal samples of each sampling were stored in sealed plastic bags and maintained at 4 °C until examination within 48 h.

**Figure 1:**
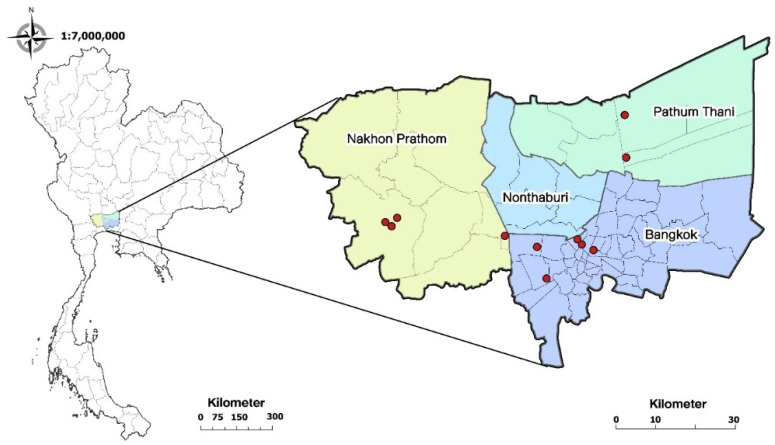
Geographical distribution of the selected 11 cat cafés for this study located in Bangkok metropolitan area, Thailand, 2017–2018.

To assess the prevalence of dermatophyte infection in cats, hair samples were collected from 198 individual cats across 11 cat cafés using Mackenzie’s toothbrush technique. Briefly, each cat was brushed thoroughly with a newly unpacked toothbrush, and any notable skin lesions such as crusts, scales, erythema, or focal alopecia were cleaned with 70% alcohol before sample collection to decrease bacterial contamination. The individual samples were stored in zipper storage bags at room temperature until the culture process commenced. The geographical distribution of sample collection is illustrated in Figure 1. This study’s protocol was approved by Chulalongkorn University Animal Care and Use Committee (Animal use protocol no. 1731065) and Chulalongkorn University Faculty of Veterinary Science Biosafety Committee (Biosafety use protocol no. 1731022).

### 2.2. Detection of Gastrointestinal Parasites

Fecal samples were examined for the presence of gastrointestinal nematode, cestode, and trematode eggs, as well as protozoan cysts and oocysts. To ensure the detection of both common and neglected endoparasites, zinc sulfate (ZnSO_4_) centrifugal flotation (1.18 specific gravity, RCI Labscan Limited, Bangkok, Thailand) and phosphate-buffered saline (PBS, Takara, Japan)-ethyl acetate (VWR International, Rosny-sous-Bois, France) centrifugal sedimentation methods were utilized [21]. Briefly, for ZnSO_4_ centrifugal flotation, around 2 g of feces was mixed with 10 mL of ZnSO_4_ flotation solution. Fecal suspension was strained and centrifuged at 800× *g* for 5 min. Then, the conical tubes were filled with flotation solution to form a positive meniscus followed by placing a cover slip for 15 min prior to microscopic examination. For PBS-ethyl acetate centrifugal sedimentation, around 2 g of feces was mixed with 10 mL 1X PBS and strained through a sieve to remove the large debris followed by pouring into a 15 mL conical tube. Then, 3 mL of ethyl acetate was added prior to vigorously shaking and centrifuged at 800× *g* for 5 min. The viscous plug on the surface of the suspension was removed, and supernatants were decanted, leaving the sediments to be sampled for microscopic examination. Slides were examined under a light microscope (Olympus CX31, Tokyo, Japan) at total magnification of 100× and 400×. To identify diagnostic stages and confirm the genus of the parasites based on the size and morphological characteristics, the standard key was used [22].

### 2.3. Detection of Dermatophytes

The protocol was adapted from previous literature, including the identification of dermatophytes using the morphology of macroconidia [19,23]. Hair samples were cultured on Sabouraud dextrose agar (SDA, DifcoTM, Becton, Dickinson and Company, Sparks, MD, USA) and Dermatophyte Test Medium (DTM, Unovetgroup, Bangkok, Thailand) at room temperature, approximately 25–30 °C, for 14 days for dermatophyte screening. Criteria for dermatophyte suspicion included the appearance of a white fluffy or radial colony on SDA and/or DTM accompanied by a color change in the DTM. After screening, suspected fungal colonies were subcultured on SDA at room temperature, approximately 25–30 °C, for 21 days to acquire a pure colony for diagnosis. The presence of dermatophytes was definitively confirmed through microscopic inspection of macroconidia having a specific shape following the direct mounting of the fungal colony using a lactophenol cotton blue staining (HiMedia Laboratories Pvt. Ltd., Mumbai, India). Slides were examined under a light microscope (Olympus CX31, Tokyo, Japan) at total magnification of 100× and 400×.

### 2.4. Data Management and Statistical Analysis

The prevalence of enteric parasites in cat cafés and dermatophytes in cats was demonstrated using descriptive statistics. Individual cat data were evaluated using binary logistic regression analysis to identify risk factors associated with the presence of dermatophytes. The result of the binary logistic regression was expressed as odds ratio (OR) and 95% confidence interval (95% CI). The dependent variables in the binary logistic regression model were positive cultures (yes/no) for *M. canis*, while the independent variables were sex, hair coat length, age, presence of skin lesion, and origin of cat. Cats were classified into short-haired and long-haired groups based on their breeds [24]. Cats affected by at least one skin lesion, such as crusts, scales, erythema, or focal alopecia were categorized as having skin lesions. The origin of each individual cat, defined as the place where cats resided before relocating to the cat café, was classified into two sources: breeding catteries and other sources (e.g., cats received from friends or adopted strays). The univariate binary logistic regression and multivariable binary logistic regression were performed. The final multivariable binary logistic regression was built by including all potential independent variables from univariable binary logistic regression due to concerns of confounding effects among independent variables. The Hosmer–Lemeshow goodness of fit test was performed for the evaluation of the fitness of the final binary logistic regression model. Statistical analyses were performed using IBM SPSS Statistics for Windows (Version 22.0. IBM Corp., Armonk, NY, USA) with a *p*-value < 0.05 considered to be significant.

## 3. Results

### 3.1. Prevalence of Endoparasites in Cats from Cat Cafés

**Figure 2:**
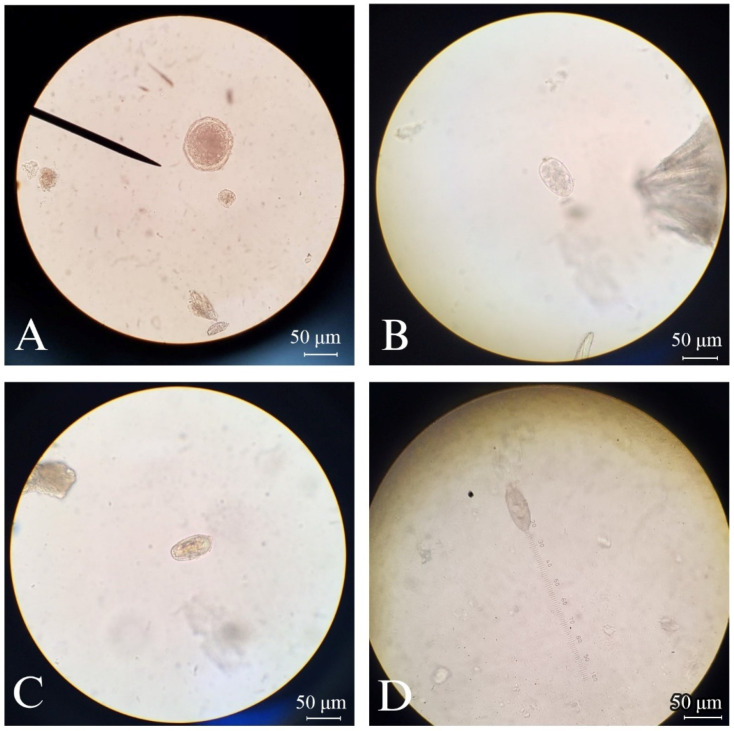
Microscopic findings of endoparasite eggs isolated from cat feces of positive cat café taken at 400× magnification: (**A**) *Toxocara* spp. (**B**) *Ancylostoma* spp. (**C**) *Physaloptera* spp. (**D**) *Eucoleus aerophilus*.

**Figure 3:**
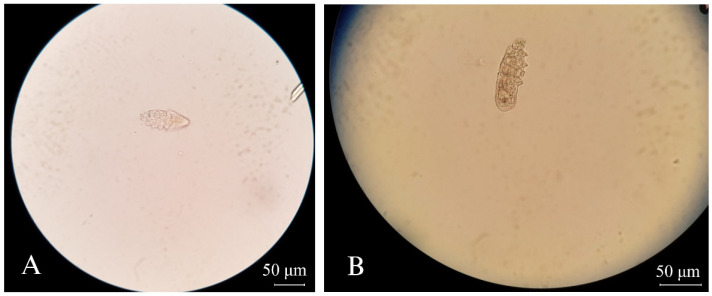
*Demodex gatoi* found in cat feces recovered by the centrifugal flotation technique, 400× magnification: (**A**) from cat café no. 03 (**B**) from cat café no. 06.

A total of 268 fecal samples were collected from 11 cat cafés located in and around the Bangkok metropolitan area. The detected prevalence of endoparasites was 18.2% (2/11) of the tested cat cafés. Figure 2 shows the helminth eggs identified, including *Toxocara* spp., *Ancylostoma* spp., *Physaloptera* spp., and *Eucoleus aerophilus*. The first three parasites were found in cat feces from the same café, café no. 5. Although centrifugal flotation was used as a qualitative technique, the record was made for the number of helminth eggs found in these samples. From four different samples, *Toxocara* eggs were found in two samples (eight and four eggs). One sample had an *Ancylostoma* egg, and another sample had a *Physaloptera* egg. Using centrifugal sedimentation, a *Toxocara* egg was found in the same sample that eight *Toxocara* eggs were found from flotation. An egg of *Eucoleus aerophilus* was found in cat café no. 9 using centrifugal sedimentation. There were no dual or multiple infections from each sample. The results of microscopic findings were contextualized with information provided by café staff regarding the history of antiparasitic product use and outdoor access for the cats. Notably, only these two helminth-positive cafés allowed cats to access outdoors, whereas other cafés exclusively kept cats indoors (Table 1). In our study, no *Giardia* cysts were detected by conventional fecal examination methods. However, *Demodex gatoi* was unexpectedly discovered through the centrifugal flotation technique from two cat cafés, specifically cafés no. 03 and 06 (Figure 3).

### 3.2. Prevalence of Dermatophytes in Cats and Risk Factors Associated with Dermatophyte Infection

**Figure 4:**
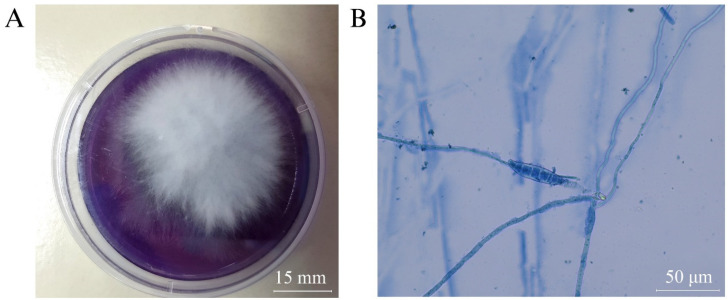
Example of dermatophytes cultured from hair samples of cats residing in cat cafés: (**A**) The growth of typical white fluffy colony on DTM media that changed to purple color after 14 days incubation. (**B**) Several spindle-shaped macroconidia of *M. canis* stained in lactophenol cotton blue from subculture on SDA for 21 days, 400× magnification.

Of the 198 hair samples from all cafés, 16.2% (32/198) of tested cats were positive for dermatophytes, with these positive cases originating from 4 of the 11 cafés (36.4%, 4/11) (Table 2). Based on SDA/DTM culture results, *Microsporum canis* was the only dermatophyte species identified in all positive subjects. A white fluffy colony on a DTM agar plate from a selected positive sample is shown in Figure 4A. Subsequent microscopic examination confirmed the presence of typical spindle-shaped *M. canis* macroconidia (Figure 4B). The risk factors correlating with positive cases are detailed in Table 3. Cats presenting with skin lesions were more likely to test positive than cats without lesions (*p* = 0.032, OR = 3.252). Interestingly, the majority of dermatophyte-positive cats were asymptomatic, as no lesions were detected (81.3%, 26/32). Notably, cats sourced from breeding catteries exhibited a significantly higher prevalence of *M. canis* infection compared with cats from nonbreeding origins, such as those received from friends or adopted strays (*p* = 0.005, OR = 18.010). However, there was no significant association between hair coat length, sex, age, and dermatophyte infection.

Additionally, during the study period, a staff member from one of the positive cat cafés reported a ringworm lesion that was subsequently identified as an infection caused by *M. canis*, the same pathogen found in approximately half of the cats in this establishment (Cat café no. 06), suggesting zoonotic transmission.

## 4. Discussion

To our knowledge, this is the first report from Thailand involving pathogen detection in cat cafes. Our findings reveal that 16.2% of cat cafes harbored endoparasites, indicating their ongoing presence as a significant, yet often asymptomatic, health concern. In this study, we detected hookworm and roundworm in cats, aligning with previous reports of endoparasite prevalence in both stray and owned cats in Thailand [12,25,26,27]. Although this cat population is not representative of all cat cafés operating in the years 2017–2018, it is important to highlight the detection of common nematodes, hookworm and roundworm, in cat café no. 05, despite monthly deworming efforts using selamectin. Several factors may contribute to the failure of preventive and control measures in this establishment, such as incomplete application for the entire clowder, suboptimal dosage, inadequate frequency, improper storage of products, or the use of expired products. Additionally, selamectin is ineffective for the treatment and control of stomach worms, *Physaloptera* spp., which were found on these premises.

The egg of a bronchial worm, *Eucoleus aerophilus*, was detected in cat café no. 09, where pyrantel was used for endoparasite control, which is ineffective against this helminth. Effective products include the topical formulation of imidacloprid 10%/moxidectin 1% [28], fipronil 8.3%, (S)-methoprene 10%, eprinomectin 0.4% and praziquantel 8.3% [29], esafoxolaner/eprinomectin/praziquantel [30], and emodepside 2.1%/praziquantel 8.6% [31].

Cats can become infected with urinary nematodes including *Pearsonema plica* and *Pearsonema feliscati*. To definitively diagnose the helminth egg collected from the litter box where the feces can be contaminated by the urine, the differentiation between *Eucoleus aerophilus* and *Pearsonema* spp. must be performed, despite no previous report on cats in Thailand. Although both of them contain bipolar plugs, the egg of *Pearsonema* spp. shows the characteristic flattened bipolar plugs, and it is passed in urine from adults living in urinary bladder mucosa. In contrast, the egg of *Eucoleus aerophilus* is oval in shape containing anastomosing ridges on the outer shell and asymmetrical, bipolar plugs [32].

In addition, *Demodex gatoi* was found in feces by centrifugal flotation in this study, paralleling previous findings [33], underscoring that fecal examination aids in diagnosing both endoparasite infections, originating from gastrointestinal and respiratory tracts, as well as ectoparasite infestations. According to Tropical Council for Companion Animal Parasites (TroCCAP) guidelines, a comprehensive deworming program coupled with a biannual parasitic infection screening program is recommended, and recent guidelines advise fecal sampling for three consecutive days to increase diagnostic sensitivity [4].

Both nematode-positive cat cafés allowed cats outdoor access. Thus, they could ingest the infective L3 hookworm larvae, which previous reports have shown to be a risk factor for hookworm infection in cats [12]. Cats can also ingest larvated eggs of roundworms, bronchial worms, and stomach worms from soil-contaminated environments. If these cats hunt for prey, they could obtain the parasites from infected paratenic hosts as well [32]. Previous literature also reported that eggs of certain parasites, including *Toxocara* spp., can persist on cats’ fur although they were not viable [34]. Therefore, strict indoor confinement could help reduce the risk of infection.

In this study, *Giardia* cysts were not found in any cat cafés using conventional fecal examination methods. To improve the sensitivity of detection, further study should employ antigen and/or molecular detection methods [35]. However, *Giardia* and *Cryptosporidium* are infectious immediately after shedding into feces [5,6], potentially contaminating café areas where food and drink are prepared, thus posing a transmission risk to customers. Recommendations could include separating the cat playing area from the dining and cooking areas, daily litter box cleaning, and handwashing before meals to minimize the risk of parasitic infection [36,37].

Published reports of the prevalence of dermatophytes in cats in Thailand are limited. The prevalence of dermatophytes in cats living in cat cafés was 16.2% (32/198), whereas the prevalences from previous studies conducted on cats from breeding farms and/or cats visiting hospitals were 28.2% (24/85) and 20.3% (28/138) in Bangkok in 2009–2010 and Chiang Mai in 2021, respectively [17,19]. These studies suggested that dermatophyte infection in cats living in Thailand is common, with around a quarter of these populations testing positive over time. *M. canis* was found in our and two other studies [17,19], but *M. gypseum* was only found in a study conducted in Bangkok [19]. The absence of *M. gypseum* from our study may be due to minimal exposure of cats to soil, the source of this geophilic dermatophyte [38].

Based on the significant association between cats in the cat café originally from breeding catteries and dermatophyte infection, this aligns with a previous study where cats from breeding farms had a higher prevalence of dermatophytes than those from hospitals [19]. Thus, veterinarians and cat owners should be more aware of dermatophyte infection in cats coming from breeding catteries. Although a study in Italy revealed no significant association between the presence of skin lesions and the prevalence of dermatophytes [39], our data and other studies in Thailand found that cats with skin lesions were more likely to have a higher prevalence than those without such lesions [17,19]. Regarding other risk factors, previous studies have indicated that dermatophyte prevalence was greater in young and long-haired cats [40,41]; however, this study, along with others, did not find a significant association between prevalence and variables such as hair coat length, age, and sex [39,42].

Since 10.5–81.3% of asymptomatic cats test positive for dermatophyte infection [17,43], including in this study, dermatophyte infection in these cats should not be excluded from diagnosis. Asymptomatic cats can serve as reservoirs to transmit arthrospores to humans and contaminate the environment [42]. Interestingly, an *M. canis* lesion was identified in a staff member from a positive cat café, suggesting a potential zoonotic transmission. Previous research also suggested an outbreak of Thai patients from asymptomatic cats residing in Bangkok [44]. Moreover, additional data suggest that zoonotic disease concerns should always be directed towards dermatophytosis in cats, as the majority of human dermatophytosis has been attributed to feline sources [45]. Thus, awareness of zoonotic transfer from asymptomatic cats should be a concern. Recommendations to control this infection in cat cafés should emphasize environmental decontamination to eliminate spore shedding, including twice-weekly cleaning and disinfection, removal of hair, and washing of target areas with a 1–10% sodium hypochlorite solution (household bleach) [38]. Isolation of infected animals is another integral component of disease control [13].

## 5. Conclusions

Zoonotic enteric nematodes and dermatophytes were identified in cat cafés in and around the Bangkok metropolitan area. Two out of eleven cat cafés tested positive for endoparasites, while four out of eleven tested positive for dermatophytes. This study highlights a significant association between outdoor access and the prevalence of endoparasites in cats. Additionally, the presence of *Microsporum canis* in both cats and a staff member underscores the zoonotic potential of dermatophyte infections within cat cafés. In response to these findings, several management and hygienic measures are recommended for cat cafés, including restricting outdoor access for cats, changing shoes prior to entering inside, and incorporating regular endoparasitic and dermatological screenings into routine health checks, as well as using broad spectrum parasiticide to consistently control both ecto- and endoparasites, even for asymptomatic animals in breeding catteries and cat cafés. Furthermore, educating staff members on the importance of early detection and isolation of infected animals can help prevent the spread of zoonotic diseases in these establishments. The implementation of a targeted infectious disease control program in cat cafés is recommended to reduce the prevalence of these zoonotic diseases.

## Figures and Tables

**Table 1:** The prevalence of cat endoparasites, the history of antiparasitic product usage, and the cat lifestyle observed in each cat café, Bangkok metropolitan area, 2017–2018.

No. of Cat Café	Total No. of Cats/ Cat Café	% Positive (Positive Samples/Tested Samples)	Type of Endoparasites (No. of Positive Sample)		Use of Antiparasitic Products		Lifestyle
			Centrifugal Flotation	Centrifugal Sedimentation	Active Ingredients	Frequency	
01	39	0% (0/61)	Not Found	Not Found	Selamectin	Every 2 months	Indoor
02	31	0% (0/35)	Not Found	Not Found	Not applied	Not applied	Indoor
03	33	0% (0/36)	Not Found	Not Found	Praziquantel + Pyrantel Embonate	Every 6 months	Indoor
04	7	0% (0/13)	Not Found	Not Found	Not applied	Not applied	Indoor
05	9	28% (4/14)	*Toxocara* spp. (1) *Toxocara* spp. (1) *Ancylostoma* spp. (1) *Physaloptera* spp. (1)	*Toxocara* spp. (1) Not Found Not Found Not Found	Selamectin	Every month	Indoor + Outdoor
06	35	0% (0/54)	Not Found	Not Found	Selamectin or Imidacloprid + moxidectin	Every 2 months	Indoor
07	7	0% (0/8)	Not Found	Not Found	Selamectin or Imidacloprid + moxidectin	Every 3-4 months	Indoor
08	16	0% (0/12)	Not Found	Not Found	Selamectin	Every 2 months	Indoor
09	12	5.9% (1/17)	Not Found	*Eucoleus aerophilus* (1)	Pyrantel Pamoate	Every year	Indoor + Outdoor
10	6	0% (0/10)	Not Found	Not Found	Not applied	Not applied	Indoor
11	3	0% (0/8)	Not Found	Not Found	Not applied	Not applied	Indoor

**Table 2:** Prevalence of dermatophyte infection in cats from each establishment, Bangkok metropolitan area, 2017–2018.

No. of Cat Café	Total No. of Cats/ Cat Café	Location of Cat Café	Type of Dermatophyte	% Positive (Positive Samples/ Tested Samples from Every Cat in Each Café)
01	39	Bangkok	*Microsporum canis*	25.6% (10/39)
02	31	Pathum Thani	Not Found	0% (0/31)
03	33	Bangkok	Not Found	0% (0/33)
04	7	Nakhon Pathom	Not Found	0% (0/7)
05	9	Bangkok	Not Found	0% (0/9)
06	35	Bangkok	*Microsporum canis*	48.6% (17/35)
07	7	Nakhon Pathom	Not Found	0% (0/7)
08	16	Bangkok	*Microsporum canis*	12.5% (2/16)
09	12	Pathum Thani	*Microsporum canis*	25.0% (3/12)
10	6	Nakhon Pathom	Not Found	0% (0/6)
11	3	Nakhon Pathom	Not Found	0% (0/3)

**Table 3:** Lists of historical data enumerating the prevalence of dermatophytes and associated risk factors in a population of 198 cats from 11 cat cafés, Bangkok metropolitan area, 2017–2018.

Variable	Categories	No. of Subject	%	Number and Percentage of Cats Positive for Dermatophytes	*p*-Value (*p*) from Binary Logistic Regression and Odds Ratio (OR)
Sex	Female	92	46.5	12/92 (13.0%)	*p* = 0.269, OR = 0.645 (CI = 0.296–1.404)
	Male	106	53.5	20/106 (18.9%)	
Hair coat length	Long-haired	40	20.2	4/40 (10.0%)	*p* = 0.243, OR = 0.516 (CI = 0.170–1.567)
	Short-haired	158	79.8	28/158 (17.7%)	
Age	<1 year	53	26.8	8/53 (15.1%)	*p* = 0.805, OR = 0.896 (CI = 0.375–2.140)
	≥1 year	145	73.2	24/145 (16.6%)	
Presence of skin lesion	Yes	17	8.6	6/17 (35.3%)	*p* = 0.032 *, OR = 3.252 (CI = 1.107–9.556)
	No	181	91.4	26/181 (14.4%)	
Origin of cat	Breeding catteries	136	68.7	31/136 (22.8%)	*p* = 0.005 *, OR = 18.010 (CI = 2.398–135.243)
	Other sources	62	31.3	1/62 (1.6%)	

*, *p*-value < 0.05.

## Data Availability

Data are contained within the article.
